# Evaluación del ensayo enhaced estradiol (eE2) en el analizador Atellica IM 1600 de Siemens

**DOI:** 10.1515/almed-2020-0002

**Published:** 2020-03-20

**Authors:** Laura Macias-Muñoz, Xavier Filella, Josep Maria Augé, Felicia A. Hanzu, Manuel Morales-Ruiz, Josep Lluis Bedini, Wladimiro Jiménez, Gregori Casals

**Affiliations:** Servicio de Bioquímica y Genética Molecular, Hospital Clínic, IDIBAPS, Barcelona, Spain; Servicio de Endocrinología, Hospital Clínic, IDIBAPS, Barcelona, Spain; Universidad de Barcelona, Barcelona, Spain; Servicio de Bioquímica y Genética Molecular, Hospital Clinic Universitari, Villarroel 170, Barcelona, 08036, Spain

**Keywords:** comparación de métodos, estradiol, evaluación de métodos, inmunoensayo

## Abstract

**Introducción:**

El estradiol en suero (E2) se emplea para el diagnóstico y/o seguimiento de una amplia variedad de patologías. El objeto del presente estudio es evaluar el ensayo enhanced estradiol (eE2) de Siemens en el sistema Atellica IM 1600 (Siemens Healthineers) y llevar a cabo una comparación de muestras con el analizador Advia Centaur XP (Siemens Healthineers).

**Métodos:**

Los coeficientes de variación (CV) intradía e interdía se evaluaron utilizando *pools* de muestras séricas y materiales de control de calidad. El límite de cuantificación se determinó mediante el empleo de seis muestras de suero con concentraciones decrecientes de E2. La linealidad fue evaluada a través del análisis de dos muestras diferentes de suero. La exactitud se determinó midiendo tres materiales de referencia certificados. Comparamos los sistemas Atellica y Centaur XP con el método de regresión de Passing–Bablok y el gráfico de Bland–Altman utilizando 119 muestras de suero con concentraciones de entre 45 y 10.059 pmol/L.

**Resultados:**

La imprecisión intradía e interdía fue <6,6%. La exactitud fue <6,0% en todos los valores de E2 evaluados excepto para la concentración de 114 pmol/L (22%). El análisis del límite de cuantificación resultó en una imprecisión interdía <20%. Se observó una alta correlación entre los valores de dilución de E2 observados y esperados (r=0,99), con un porcentaje de recuperación que oscila entre el 77 y el 93%. El estudio comparativo mostró un buen nivel de concordancia, sin hallarse un sesgo significativo.

**Conclusiones:**

Este estudio indica que el ensayo eE2 tiene una imprecisión y una exactitud aceptables, mostrando una buena correlación con el sistema Centaur XP.

## Introducción

El estradiol (17β-estradiol) (E2) es el estrógeno natural más potente y más frecuentemente analizado en la práctica clínica e investigación. La cuantificación en suero de esta hormona esteroide de 18 átomos de carbono aporta información valiosa en el contexto de numerosas patologías endocrinológicas y ginecológicas. De este modo, el análisis de las concentraciones de E2 resulta útil en la reproducción asistida para monitorizar el desarrollo folicular, así como en la evaluación clínica del hipogonadismo, hirsutismo, síndrome de ovario poliquístico, amenorrea, infertilidad femenina, menopausia, tumores gonadales, feminización en hombres o para la monitorización de pacientes con cáncer de mama que estén recibiendo terapia con inhibidores de la aromatasa, entre otras patologías [1]. Los niveles fisiológicos de E2 son bajos (con frecuencia <184 pmol/L) en el suero de hombres, niños y mujeres posmenopáusicas, aunque pueden variar considerablemente en las mujeres premenopáusicas (110–2.937 pmol/L) [2]. Por otro lado, las diferencias esperadas en las concentraciones de E2 en suero son mayores, abarcando hasta cinco órdenes de magnitud si se tienen en cuenta la diversidad de sus aplicaciones clínicas. Por ejemplo, las concentraciones séricas de E2 pueden alcanzar los 36.713 pmol/L en las determinaciones realizadas en los programas de fertilización *in vitro*, mientras que la terapia con inhibidores de la aromatasa pueden reducir las concentraciones séricas de E2 hasta los 3.7 pmol/L [3]**.** En resumen, actualmente se utiliza una amplia variedad de intervalos de referencia de E2 sérico en diferentes contextos clínicos, lo cual requiere el uso de sistemas analíticos de alta calidad. En este sentido, la evaluación de la capacidad analítica de un test de E2 es esencial para determinar su fiabilidad a la hora de responder a cuestiones clínicas o de investigación [2].

Se han desarrollado inmunoensayos automáticos directos (sin extracción) de alto rendimiento para hacer frente al creciente número de peticiones de determinación de las concentraciones de E2, y que pueden ser utilizados en la mayoría de laboratorios clínicos. No obstante, numerosos estudios indican de forma consistente que la especifidad y exactitud de los inmunoensayos directos a concentraciones bajas son insatisfactorias [4], [5], [6], [7], [8], [9], [10], [11]. Recientemente se han lanzado al mercado dos nuevos analizadores (Atellica IM 1300 y Atellica IM 1600, Siemens Healthineers, Erlangen, Alemania), y su uso en el laboratorio clínico está creciendo. A este respecto, evaluamos el rendimiento analítico del ensayo enhanced Estradiol (eE2) de Siemens en el sistema Atellica IM 1600 (Siemens Healthineers, Erlangen, Alemania). Concretamente, analizamos la imprecisión, exactitud, límite de cuantificación y linealidad del método analítico. Así mismo, comparamos su rendimiento con otro método, con especial atención a concentraciones bajas E2.

## Materiales y métodos

### Principio del ensayo e instrumento

El ensayo eE2 de Atellica IM es un inmunoensayo competitivo quimioluminiscente, en el que se emplea un anticuerpo de oveja monoclonal para el estradiol marcado con éster de acridinio. En primer lugar, el E2 endógeno es liberado de sus proteínas ligantes por un agente liberador. A continuación, se añade el anticuerpo marcado para que se una al E2 disponible. También se añade a la reacción un conjugado de captura de E2 acoplado a partículas de látex magnéticas, que compite con el E2 endógeno por unirse al anticuerpo marcado. Tras el lavado, se añade ácido y base para iniciar una reacción quimioluminiscente. Existe una relación inversamente proporcional entre la cantidad de E2 en la muestra de suero y la cantidad de unidades relativas de luz (URL) detectadas por el instrumento. Las mediciones se realizaron con el analizador IM 1600 de Atellica. El primer resultado se arroja a los 18 minutos, y se realizan cuantificaciones sucesivas en incrementos de 20 segundos. El volumen de muestra requerido es de 80 μL. Los límites de detección y cuantificación indicados por el fabricante son 43 y 70 pmol/L, respectivamente. El intervalo de medida abarca entre los 43 y 11.014 pmol/L.

### Evaluación del método

#### Imprecisión y exactitud

La imprecisión intradía se calculó analizando alícuotas de dos pools de suero, tres niveles de material de control comerciales (Liquicheck Immunoassay Plus, Bio-Rad, Irvine, CA) y tres materiales de referencia certificados (BCR-576, BCR-577, and BCR-578, Joint Research Center, Comisión Europea). Se analizaron cinco réplicas de cada muestra en el mismo día. La imprecisión interdía se calculó empleando las mismas cinco muestras en cinco días distintos. También se utilizaron los materiales de referencia certificados BCR-576, BCR-577 y BCR-578 para evaluar la exactitud intradía e interdía del ensayo. Los requisitos aceptables de imprecisión y exactitud se establecieron a partir de las especificaciones deseables basadas en la variabilidad biológica [12].

#### Límite de cuantificación

El límite de cuantificación se determinó analizando seis pools de suero con una concentración media de E2 de entre 52,9 y 223 pmol/L, que se midieron ocho veces en cinco días diferentes. Actualmente no existe consenso sobre el nivel de imprecisión aceptable para la cuantificación clínica de E2. El límite de cuantificación se estableció como la concentración que presentaba un coeficiente de variación (CV) del 20%, valor empleado habitualmente [3], [13].

#### Linealidad del método

Para el estudio de linealidad se emplearon dos muestras de suero con diferentes concentraciones de E2 (1.957 y 3.748 pmol/L). Se realizaron diluciones seriadas de cada muestra con el diluyente específico del ensayo para obtener 1/2, 1/5, y 1/10 de la concentración original (reactivo auxiliar Atellica IM eE2 DIL ReadyPack).

#### Comparación de métodos

Se analizaron 119 muestras de suero con concentraciones entre 44 y 10.059 pmol/L simultáneamente en los sistemas Atellica IM 1600 y ADVIA Centaur XP (Siemens Healthineers).

También se analizaron con Atellica IM 1600 un total de 29 muestras de suero con concentraciones bajas de E2 medidas previamente con el ELISA EIA-4399 (DRG International, Inc, Springfield, EE,UU) (n=4 muestras <11pmol/L, n=16 entre 11–43 pmol/L, n=5 entre 43–73 pmol/L y n=4 entre 73–110 pmol/L). Este también es un inmunoensayo directo, que cuenta con una incubación prolongada de horas y un menor rango analítico de 0–734 pmol/L que permite un límite de detección (LOD) inferior según el fabricante (5,1 pmol/L).

### Análisis estadístico

El análisis estadístico se realizó con Microsoft Excel y R Studio (Versión 1,1,463 – © 2009–2018 RStudio, Inc). La imprecisión en cada nivel de concentración y el límite de cuantificación se indican como coeficientes de variación (%CV). La exactitud se calculó como la diferencia entre las concentraciones de E2 y las concentraciones nominales indicadas por el fabricante y se expresó en porcentaje. La linealidad de las diluciones seriadas a diferentes concentraciones de E2 se determinó mediante análisis de regresión lineal y las recuperaciones se calcularon utilizando la fórmula [(concentración observada – concentración esperada)/concentración esperada] × 100. Se asumió que el método se comportaba linealmente si el coeficiente de correlación era superior a 0,95. Por último, se utilizó el método de regresión de Passing–Bablok para evaluar las diferencias entre los dos métodos analíticos. Se construyeron gráficos de Bland–Altman para evaluar el sesgo sistemático entre los dos métodos.

El presente estudio se realizó cumpliendo con los principios de la Declaración de Helsinki en relación con la investigación ética en seres humanos.

## Resultados

### Imprecisión y exactitud

En la Tabla 1 se resumen los valores obtenidos en el estudio de imprecisión. La imprecisión intradía e interdía fue <5,0% y<7,0% respectivamente en todas las concentraciones de E2 evaluadas (entre 175 y 3.848 pmol/L). La Tabla 2 muestra los valores de exactitud e imprecisión para los tres materiales de referencia certificados. La imprecisión fue <6,0% a todas las concentraciones evaluadas. La exactitud fue <7,0% para concentraciones de E2 intermedias (690 pmol/L) y altas (1.340 pmol/L), y 22% (exactitud interdía) a concentraciones bajas (114 pmol/L).

**Tabla 1:** Resultado del estudio de imprecisión expresado como coeficiente de variación realizado con controles de calidad y pools de suero.

	Media	Imprecisión, %	
	Estradiol	Intradía	Interdía
	pmol/L	(n=5)	(n=5)
QC-1	175	4,0	6,5
QC-2	1.336	1,8	4,1
QC-3	3.848	1,2	1,0
Pool de muestras 1	903	3,5	6,6
Pool de muestras pool 2	3.301	2,4	2,2

QC, control de calidad; n, número de mediciones.

**Tabla 2:** Resultados del estudio de imprecisión y exactitud expresados como coeficiente de variación realizado en tres materiales de referencia certificados.

	Resultado teórico	Imprecisión, %		Exactitud, %	
	Estradiol	Intradía	Interdía	Intradía	Interdía
	pmol/L	(n=5)	(n=5)	(n=5)	(n=5)
BCR-576	114	5,9	4,6	29	22
BCR-577	690	1,5	1,8	0,7	0,0
BCR-578	1.340	1,4	2,1	6,9	5,2

n, número de mediciones.

### Límite de cuantificación

**Figura 1:**
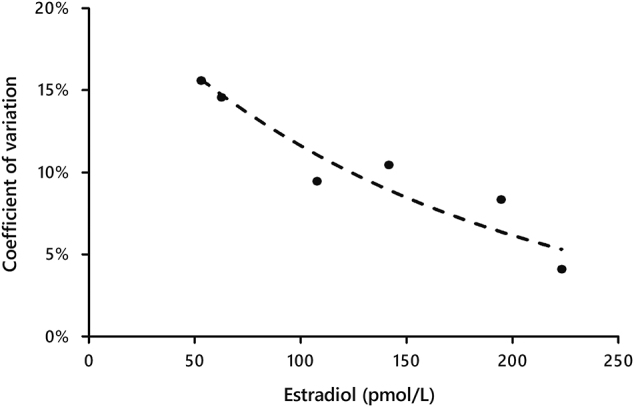
Límite de cuantificación del ensayo eE2 de Atellica IM 160 338 × 190 mm (96 × 96 DPI).

La imprecisión del ensayo también se evaluó en concentraciones decrecientes de E2 empleando muestras de suero. Todas las muestras de suero mostraron una imprecisión interdía <20%, siendo la concentración más baja evaluada 53 pmol/L. La concentración más baja de E2 que mostró un CV<10% fue 125 pmol/L (Figura 1).

### Linealidad

**Figura 2:**
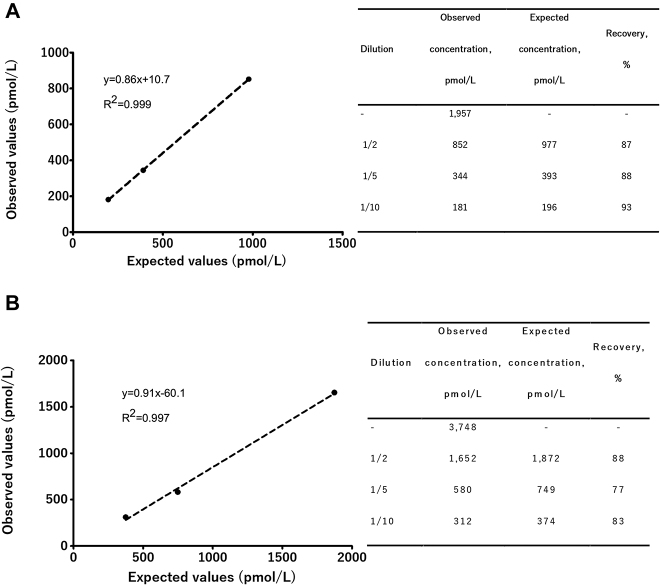
Estudio de linealidad del ensayo eE2 de Atellica IM 1600 realizado a partir de dos muestras de suero de 1.957 pmol/L (A) y 3.748 pmol/L (B).

Se obtuvo un elevado coeficiente de correlación entre los valores observados y los esperados (r>0,99) en los dos grupos de diluciones seriadas de E2. El porcentaje de recuperación medio obtenido en las diluciones seriadas A y B fue de 89 y 83% respectivamente (Figura 2).

### Comparación de métodos

**Figura 3:**
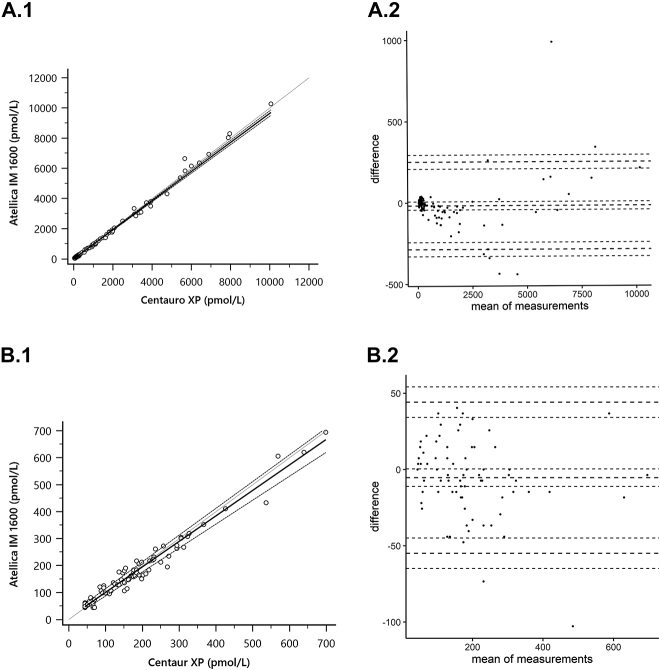
Comparación de métodos. Análisis de regresión de Passing–Bablok y Bland–Altman comparando los ensayos eE2 de Atellica IM 1600 y eE2 de Advia Centaur XP. (A.1) Análisis de regresión de Passing–Bablok abarcando el intervalo completo de concentraciones de E2 (n=119). (A.2) Gráfico de Bland–Altman del intervalo completo de concentraciones de E2 (n=119). (B.1) Análisis de regresión de Passing–Bablok de concentraciones de E2 <734 pmol/L (n=119). (B.2) Gráfico de Bland–Altman de concentraciones de E2 <734 pmol/L (n=76).

En la Figura 3 se muestra la comparación de los resultados obtenidos en el análisis de las muestras de suero en Atellica IM 1600 y ADVIA Centaur XP. Ambos analizadores mostraron una buena intercambiabilidad en todo el intervalo (R^2^=0,99) y a concentraciones de E2 <734 pmol/L (R^2^=0,96). La ecuación de regresión de Passing–Bablok fue: Atellica = 0,96 (IC95% 0,95–0,98) × Centaur + 2,8 (IC95% −4,9–8,7) para todo el intervalo y Atellica = 0,95 (IC95% 0,89–0,99) × Centaur + 5,9 (IC95% −2.76–14) para concentraciones inferiores a 734 pmol/L. El sesgo entre los dos métodos fue de 18 pmol/L (IC95% −43–6,9) para todo el intervalo y −5,3 pmol/L (IC95% −11–0,41) para concentraciones inferiores a 734 pmol/L.

En la Tabla 3 se muestran los resultados de la comparación realizada entre Atellica IM 1600 y DRG Sensitive ELISA. Las cuatro muestras de suero con concentración <11 pmol/L según DRG Sensitive ELISA también presentaron resultados inferiores al límite de detección en Atellica IM 1600 (< 43,3 pmol/L). Once de las 16 muestras con valores de E2 entre 11,0 y 43,3 pmol/L según DRG Sensitive ELISA presentaron también concentraciones indetectables (<43,3 pmol/L) en Atellica IM 1600. Así mismo, ocho de las nueve muestras con valores superiores a 43,4 pmol/L según DRG Sensitive ELISA mostraron niveles detectables por Atellica IM 1600 (>43,3 pmol/L)

**Tabla 3:** Resultados del estudio de comparación realizado entre Atellica IM 1600 y DRG Sensitive ELISA.

DRG Sensitive ELISA pmol/L	n	Atellica IM 1600 pmol/L	n	Concordancia, %
<11	4	<43	4	100%
11–43	16	≤43	11	69%
		>43	5	
≥43	9	≥43	8	89%
		<43	1	

n, número de mediciones.

## Discusión

En este estudio, evaluamos el rendimiento analítico del ensayo eE2 en Atellica IM 1600. Los inmunoensayos directos de E2 ofrecen multitud de ventajas tales como que se comercializan como ensayos automatizados, lo cual evita una laboriosa extracción de las muestras, y su alto rendimiento y rápido tiempo de respuesta. Contar con un método de cuantificación que abarque un amplio intervalo dinámico de resultados es esencial para los laboratorios diagnósticos, especialmente aquellos en los que se realizan pruebas de fertilidad.

En términos generales, el rendimiento del ensayo ha sido adecuado, mostrando una buena correlación lineal, con una imprecisión dentro del intervalo clínico habitual. De acuerdo con Stanczyk et al. [2], nos centramos en evaluar la imprecisión y exactitud en valores de E2 <3,671 pmol/L. Obtuvimos una imprecisión intradía (CV entre 1,8–4,0%) e interdía (CV entre 1,0 y 6,6%) satisfactorias en los cinco niveles de concentración evaluados tanto en los controles de calidad como en los *pools* de suero, cumpliendo con la especificicación deseable de imprecisión basada en la variabilidad biológica (<11%) [12]. Los valores de imprecisión obtenidos son similares a los indicados por el fabricante y son comparables a los descritos previamente para el ensayo de eE2 en Advia Centaur [14]. La exactitud del ensayo se evaluó a concentraciones de 114, 690 y 1,340 pmol/L empleando materiales de referencia certificados. Los resultados obtenidos con Atellica IM eE2 se compararon con los valores certificados aportados por el fabricante y que fueron determinado con un método de referencia basado en cromatografía de gases y espectrometría de masas con dilución isotópica (GC-MS). La exactitud fue buena (<6,9%) y cumplió con los requisitos de calidad deseables basados en las especificaciones de variabilidad biológica (<8,3%) [12] a concentraciones de 690 y 1,340 pmol/L. Sin embargo, la exactitud a concentraciones bajas (114 pmol/L) fue del 28%, no cumpliendo con los requisitos de calidad. Esto podría ser explicado por las dificultades que presentan los inmunoensayos directos para detectar de forma fiable concentraciones bajas de E2, debido a la reacción cruzada con otras moléculas esteroides o elementos interferentes que pueden provocar un sesgo significativo [6], [7]. De hecho, Ketha et al. [13] describieron que el análisis de datos realizado en un estudio del College of American Pathologists mostraba que una concentración de E2 de 106 pmol/L medida con LC–MS/MS era sobreestimada (40–300%) por todas las plataformas de inmunoensayo evaluadas. En este estudio, diferentes plataformas de ADVIA Centaur presentaron un sesgo positivo del 40% aproximadamente.

Las mediciones repetidas de una muestra libre de E2 (diluyente del ensayo eE2, n=10) no dieron resultados detectables. Sin embargo, la determinación del LOD no fue posible ya que la plataforma Atellica IM 1600 no informa valores inferiores a 43 pmol/L. En su lugar, pudimos evaluar el límite de cuantificación como el CV interensayo en niveles decrecientes de E2 en suero, obteniendo un CV<20% a 53 pmol/L y un CV<10% a 125 pmol/L. Esto indica que, aunquela exactitud del ensayo es deficiente a concentraciones bajas, la imprecisión es buena en todo el rangode ensayo, incluyendo las concentraciones bajas. Esto concuerda con los resultados obtenidos por Chen et al. para el ensayo eE2 de ADVIA Centaur [14].

En resumen, los resultados obtenidos indican que el método es adecuado para la cuantificación de concentraciones de E2 superiores a 43 pmol/L. Sin embargo, tal como ocurre con otros inmunoensayos directos, este ensayo no resulta útil a la hora de evaluar concentraciones muy bajas de E2 en suero, como las esperadas en mujeres posmenopáusicas tratadas con inhibidores de la aromatasa para el tratamiento del cáncer de mama. En este escenario, es necesario mejorar los métodos existentes para que estos sean capaces de distinguir concentraciones suprimidas (inferiores a 3,7 pmol/L) de aquellas observadas pretratamiento (habitualmente entre 37 y 55 pmol/L) [3]. La espectrometría de masas y los radioinmunoensayos indirectos son las alternativas propuestas para lograr alcanzar este nivel de sensibilidad analítica. La estandarización de los inmunoensayos de estradiol es también necesaria para cuantificar mejor los niveles de E2 en suero en situaciones clínicas específicas.

La comparación de muestras entre Atellica IM 1600 y ADVIA Centaur XP eE2 abarcó todos los niveles de concentración, incluyendo aquellos <734 pmol/L, que son los valores más frecuentes en nuestra rutina diaria de trabajo. No se detectó sesgo significativo en todo el rango de concentración evaluado ni a concentraciones <734 pmol/L, observándose un alto nivel de concordancia entre ambos ensayos. Finalmente, Atellica IM 1600 y DRG Sensitive ELISA también mostraron un buen nivel de concordancia, y la mayoría de las concentraciones inferiores al LOD de Atellica (<43 pmol/L) también fueron <43 pmol/L por DRG.

En resumen, el ensayo eE2 de Atellica IM 1600 posee una imprecisión y una exactitud aceptables por encima del límite de detección, y ha demostrado un buen nivel de concordancia con ADVIA Centaur XP.
